# A mechanistic basis for amplification differences between samples and between genome regions

**DOI:** 10.1186/1471-2164-13-455

**Published:** 2012-09-05

**Authors:** Colin D Veal, Peter J Freeman, Kevin Jacobs, Owen Lancaster, Stéphane Jamain, Marion Leboyer, Demetrius Albanes, Reshma R Vaghela, Ivo Gut, Stephen J Chanock, Anthony J Brookes

**Affiliations:** 1 Department of Genetics, University of Leicester, Leicester, LE1 7RH, UK; 2 Division of Cancer Epidemiology and Genetics, National Cancer Institute, Bethesda, 20892-7335, USA; 3 Core Genotyping Facility, National Cancer Institute, SAIC-Frederick Inc., Gaithersburg, MD, USA; 4 INSERM U 955, Psychiatrie Génétique, Créteil, 94000, France; 5 Université Paris Est, Faculté de Médecine, Créteil,, 94000, France; 6 AP-HP, Hôpital H. Mondor – A. Chenevier, Département de Psychiatrie, Créteil,94000, France; 7 Centre Nacional d'Analisi Genomica, Barcelona, 08028, Spain

**Keywords:** DNA amplification, DNA denaturation, C + G, Illumina infinium

## Abstract

**Background:**

For many analytical methods the efficiency of DNA amplification varies across the genome and between samples. The most affected genome regions tend to correlate with high C + G content, however this relationship is complex and does not explain why the direction and magnitude of effects varies considerably between samples.

**Results:**

Here, we provide evidence that sequence elements that are particularly high in C + G content can remain annealed even when aggressive melting conditions are applied. In turn, this behavior creates broader ‘Thermodynamically Ultra-Fastened’ (TUF) regions characterized by incomplete denaturation of the two DNA strands, so reducing amplification efficiency throughout these domains.

**Conclusions:**

This model provides a mechanistic explanation for why some genome regions are particularly difficult to amplify and assay in many procedures, and importantly it also explains inter-sample variability of this behavior. That is, DNA samples of varying quality will carry more or fewer nicks and breaks, and hence their intact TUF regions will have different lengths and so be differentially affected by this amplification suppression mechanism – with ‘higher’ quality DNAs being the most vulnerable. A major practical consequence of this is that inter-region and inter-sample variability can be largely overcome by employing routine fragmentation methods (e.g. sonication or restriction enzyme digestion) prior to sample amplification.

## Background

The fact that amplification methods vary in efficiency across the genome has often been noted, for example in whole genome amplification (WGA), next generation sequencing, genome wide SNP genotyping, and PCR [1-5]. Difficult to assay regions are somewhat correlated with high C + G content [1,6-10], but this relationship is complex, DNA sample dependent, and incompletely understood. Regions of high C + G content tend to resist the essential DNA denaturation step at the initiation of nearly all DNA amplification protocols, though it is assumed that this effect will not be so extreme as to completely prevent DNA strand separation. However, this assumption may be incorrect. In DNA melting studies in the early 1970s, select human genome DNA fragments were seen to remain double stranded under extreme denaturing conditions [11,12]. The nature of these challenging sequences has not yet been determined, and today most investigators are probably unaware of the early reports.

Here, we investigate a number of genomic regions that across several samples produce low intensity hybridization in Illumina Infinium genotyping. We find that a major factor that can influence such regions are intervals of high C + G content that do not denature efficiently under routinely used conditions. These intervals cause connected DNA sequences to rapidly re-anneal and prevent access to primers or probes. The effects of this in PCR could be completely ameliorated by enzymatic separation of the high C + G interval and the assay target. We postulate that inter-sample variability is due to the amount and random distribution of nicking within a DNA sample which acts to separate these difficult to denature sequences from other DNA, and that highly intact DNAs will suffer the most. We provide optimized PCR protocols and suggest that DNA is pretreated by either sonication or restriction enzyme digestion prior to amplification steps in methods.

## Results and discussion

### Testing DNA melting using southern blot hybridisation

**Figure 1:**
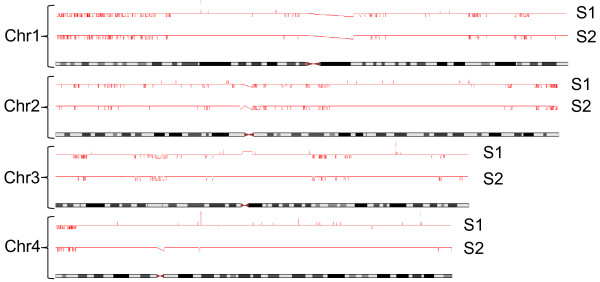
**Correlated weak signal regions in Illumina Infinium array data.** Signal intensity data from Illumina genotyping arrays (expressed as copy number counts per SNP), are shown in red above four example chromosome ideograms for two independently processed DNAs (S1 and S2), the data for which failed standard quality control checks. Whereas most markers can be seen to have produced normal strength signals (inferred diploid copy number of 2, indicated by the most prominent horizontal row of data points), many other markers produced far weaker signals (inferred copy number of one, indicated by the row of data points plotted one step lower), and these weak signal regions are highly correlated between the two samples (and between many others not shown here).

**Figure 2:**
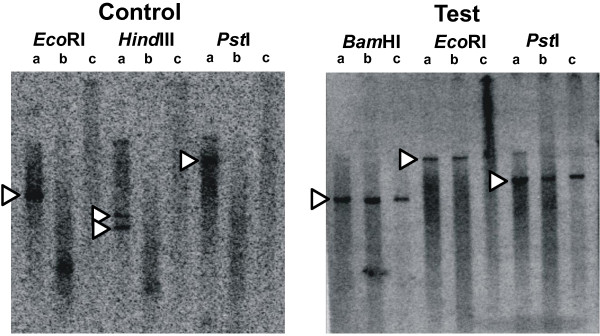
**Southern blot data showing DNA fragments that resist denaturation.** Data is shown for Southern blots in which freshly prepared genomic DNAs were cut with the indicated restriction enzymes and processed as normal (‘a’ tracks), or heated for one minute in water at 100°C and snap cooled on ice prior to gel electrophoresis (‘b’ tracks), or similarly heated and cooled before restriction enzyme digestion and electrophoresis (‘c’ tracks). Arrow heads indicate the expected position of Southern blot bands. The ‘Control’ probe (PSCDBP, Table 1), which is from a genome region that gives consistently strong Illumina Infinium signals, produces no bands in any heated sample. In contrast, the ‘Test’ probe (CAPN10, Table 1), which originates from a genome region that tends to give weak Illumina Infinium signals, produces strong bands in all the tracks, indicating the detected genomic fragments are not effectively denatured by the conditions applied prior to running on the agarose gel. Equivalent results were produced for denaturation attempts involving heating at 37°C for 10 minutes in 0.32 M NaOH, followed by pH neutralisation (data not shown).

To explore the interplay between DNA melting and difficult to assay genome regions, we examined large scale Illumina Infinium SNP array datasets (from genome wide association analyses) and identified genomic regions within which SNPs consistently gave weak intensity signals in the poorest performing samples (example given in Figure 1). We herein refer to these as ‘weak Illumina signal’ regions. Single copy DNA probes were constructed for ‘weak Illumina signal’ and control ‘normal Illumina signal’ regions on the long arm of Chr 2 (Table 1), to be hybridized on to Southern blots. These blots employed freshly prepared high quality genomic DNAs, and each sample was divided into four aliquots so that we could differentially process them by temperature or alkaline denaturation before or after restriction enzyme digestion. One would expect the denatured DNA to migrate differently to dsDNA and not give bands of expected restriction fragment sizes upon hybridization with the single copy probes, but any sequences that had fully resisted the denaturation treatments would give such bands. Example results are shown in Figure 2. Three of three ‘normal Illumina signal’ region probes produced the expected ‘no band’ outcome, whereas two of the three ‘weak Illumina signal’ region probes generated bands from the denatured samples indicating that these latter regions are generally difficult to denature.

**Table 1:** Primers and co-ordinates for all PCR amplicons and Probes (hg18, GRCh36)

**ID**	**Forward primer sequence**	**Reverse primer sequence**	**Co-ordinates (GRCh36)**	**Length**	**Type**
HDLBP	GAGCTCATCCTCCACTTGGG	GAACTTGGTGAGAAGTGCGG	chr2:241,855,412-241,860,011	4600	TUF
HDAC4	AGGTGCTAGATTTGGACGGG	GTGTGTGTTAGGGGGTCAGG	chr2:239,860,758-239,863,570	2813	TUF
CAPN10	ATCTGGCTACAGGCATGGGC	GAGAGCCCAGAAGTTCCAGC	chr2:241,173,030-241,175,779	2750	TUF
PSCDBP	GAGGCAATCACATGAGCAGG	CTGCTAAGTGGATGAATGGTGG	chr2:158,003,567-158,006,035	2469	Non-TUF
MARCH7	GGGAAATATGGGTTGGGAAACTG	ATGGTCTCCGTCTTCTTCGG	chr2:160,329,568-160,332,075	2508	Non-TUF
RBMS1	AGTAAGGAGATGAGGGGTGG	ACAGGTTTTGGTGGGAGAGG	chr2:160,889,938-160,892,713	2776	Non-TUF
2n13	GCAGACTAATGGGGATGAGG	GCCTATCTGGAAAAATAGAC	chr2:241,151,005-241,151,733	729	TUF
			chr13:31,949,825-31,950,179	355	Non-TUF
8n6	TTGAGTCAGCCACAGAGG	CCTGGTGACAGAATGACC	chr8:142,286,472-142,286,937	466	TUF
			chr6:89,689,978-89,690,367	390	Non-TUF
8n3	GCTTCATCCAGCTTCAACC	AGCAAAGTGACACTCAGTGC	chr8:145,234,022-145,234,464	443	TUF
			chr3:134,693,119-134,693,550	432	Non-TUF
2n1	CACCCCAGTGAGTAAGCTGC	AGGGTGATCGCTTCTGACC	chr2:241,707,017-241,707,258	242	TUF
			chr1:37,721,785-37,722,026	242	Non-TUF
5nX	ATCTAGGCTCAGGAGAGAG	TAAACATCTTAAAATGGCCT	chr5:179,593,910-179,594,270	361	TUF
			chrX:63,694,585-63,694,959	375	Non-TUF
9n14	CAGAGAGCAACCTGGCTC	CTGCCTCCTTGTTTGGC	chr9:139,562,111-139,562,372	262	TUF
			chr14:94,217,995-94,218,256	262	Non-TUF

### Reduced PCR amplification efficiency assessed by PRT

We examined normal and ‘weak Illumina signal’ regions using the Paralogue Ratio Test (PRT) [13,14]. Standard PRT, which is a powerful technique to genotype copy number variation, employs a single pair of PCR primers to co-amplify a ‘test’ locus (whose copy number is being assessed) and a ‘reference’ locus (a stable single copy sequence) in a single PCR reaction. The two amplicons are distinguished by size, and their relative product amounts used to determine the test locus copy number. We adapted this concept to co-amplify single copy sequences from normal and ‘weak Illumina signal’ regions. This allowed the comparison of their relative amplification efficiencies in the same PCR reaction with identical conditions and DNA template concentration. Importantly, the ‘test’ and ‘reference’ amplicons employed for six assay designs created for these experiments had similar and not unusually high C + G content (average values of 56.8 and 51.0% C + G respectively). In all six assays, the ‘reference’ amplicon (i.e., the product amplified from the assay’s normal Illumina signal region) produced a strong band, whereas its partnered ‘test’ amplicon produced a weaker band (typically 10-50% of the strength of the reference), indicating a reduced PCR efficiency for ‘weak Illumina signal’ regions.

### Enhancing denaturing conditions improves amplification

**Figure 3:**
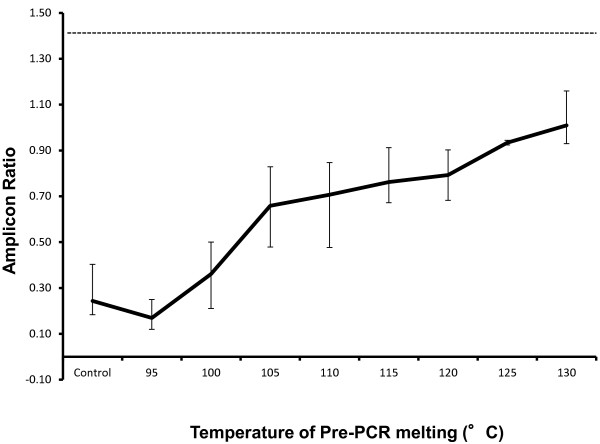
**Pre-heating samples to improve PCR amplification efficiency.** This graph plots the test:reference product ratio generated by PRT assay ‘2n13’ (Y-axis), amplicon sizes 729 bp and 355 bp, against temperatures used to pre-heat the input genomic DNAs (X-axis). After heating, samples were snap cooled on ice before adding them to the PRT reaction mix. Each condition was run on 2–4 samples in duplicate, and maximum and minimum ratios are plotted as error bars. The ‘Control’ ratio at the start of the chart indicates the ratio produced by amplifying non-heated input DNA, and the dotted horizontal line at 1.43 indicates the ratio that would be produced if the slightly different sized test and reference amplicons amplified with exactly equal efficiency.

The above data are consistent with the hypothesis that ‘weak Illumina signal’ regions are refractory to amplification and analysis because they are difficult to denature. To promote DNA denaturation in the PRT assays, and thereby increase the amplification efficiency of the ‘weak Illumina signal’ loci, we tried the following standard denaturing enhancers; including Dimethyl sulphoxide (DMSO) at up to 50% [15,16]; adding Single Stranded Binding Protein [17]; increasing the PCR denaturing temperature to 98°C. These strategies all helped to improve amplification efficiency, but none of these remedies enabled a full strength intensity gel band to be produced for any of the ‘weak Illumina signal’ loci. Adding Betaine [16,18] was more effective, but only at very high concentrations (i.e., at 1.5-2.0 M), with the downside of causing overall amplification efficiencies to drop considerably. Most effective was denaturing the input DNAs, and snap cooling on ice, prior to inclusion in the PCRs. However, to significantly improve the amplification efficiencies (Figure 3), it was necessary to heat the samples to 130°C in water for 1 minute (longer or hotter reduced PCR efficiency presumably due to excessive DNA hydrolysis).

### Regions of high C + G serve as nuclei for rapid re-annealing of neighboring DNA sequences

**Figure 4:**
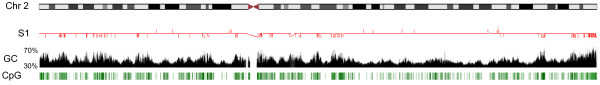
**Illumina Infinium weak signals regions aligned with CpG and C + G maps.** This image uses chromosome 2 to provide typical evidence of the degree of correlation between copy number inferences per SNP for samples that genotyped poorly on Illumina Infinium arrays (first data row below the ideogram), long-range averaged C + G content on a scale of 30-70% (middle data rows, data from UCSC genome browser), and the location of CpG islands (bottom data rows, data from UCSC genome browser). Weak Illumina signal regions are not simply correlated with CpG islands, nor with generally high C + G content, but only with regions containing the highest peaks of C + G content.

Cumulatively, these findings show that ‘weak Illumina signal’ regions are particularly resistant to DNA denaturation under standard conditions. This is true, even when tested PCR amplicons themselves are not particularly C + G rich or unusual in any apparent way (in fact, for two PRTs the test and reference were almost identical). The implication of this is that locally something other than C + G content of the target sequence is hindering DNA strand separation. Direct visualization of genome features represented as tracks on the UCSC genome browser suggests this may have something to do with the very highest peaks of C + G rich sequence coincident with particularly dense clustering of CpG islands (Figure 4). A possible mechanism could then entail localized regions of extreme C + G content remaining duplexed during standard DNA denaturation procedures, and in so doing they would prevent their flanking sequences - that are melted - from diffusing away from each other. As such, these neighboring strands will be able to quickly re-anneal, following zero-order kinetics, as soon as non-denaturing conditions are re-established [19]. We refer to domains affected by this proposed phenomenon as "Thermodynamically Ultra Fastened" (TUF) regions.

**Figure 5:**
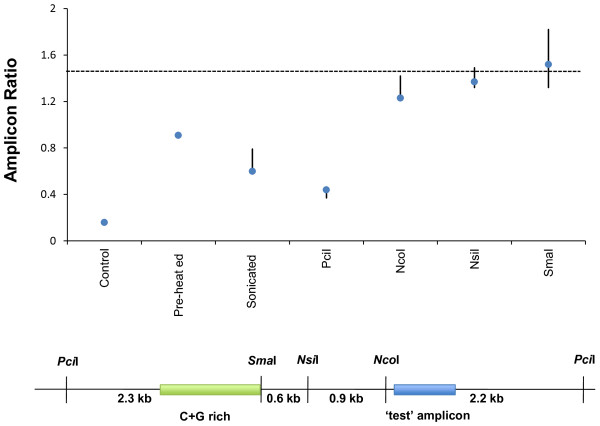
**TUF (C + G rich) sequences impair the analysis of neighbouring DNA regions.** The lower image shows a restriction map surrounding the ‘test’ fragment of PRT assay A2n13, including a 1100 bp region with 73% C + G content (green box). The graph above shows average test:reference product ratios that were run in triplicate, with maximum and minimum plotted as error bars. ‘Control’ indicates the use of undigested DNA. Remaining columns show the ratios produced upon pre-digesting with the indicated restriction enzymes. The absolute degree of reference fragment amplification did not vary significantly across these treatments. Treatments that break the DNA to physically separate the test fragment from the C + G rich sequence clearly provide the best improvement in test fragment amplification efficiency. This reaches 1.43, which is the theoretical maximum assuming exactly equal molar amplification of test and reference amplicons (as indicated by the dotted line).

To test the TUF hypothesis, we started by looking for localized, highly C + G rich DNA elements in the immediate vicinity of the ‘weak Illumina signal’ region amplicons for the six PRT assays. Such elements were clearly present in five cases. We then targeted one particular assay (‘2n13’: for which the ‘test’ and ‘reference’ efficiencies were most different) and digested the template DNA with various restriction enzymes before running the PRT. DNA amplification was seen to be problematic only when the ‘test’ amplicon was located in the same DNA fragment as the high C + G element (Figure 5). In fact, the amplification efficiency was fully restored when the ‘test’ amplicon was separated from the high C + G element, a finding consistent with the TUF hypothesis.

### Genome wide patterns of TUF

**Figure 6:**
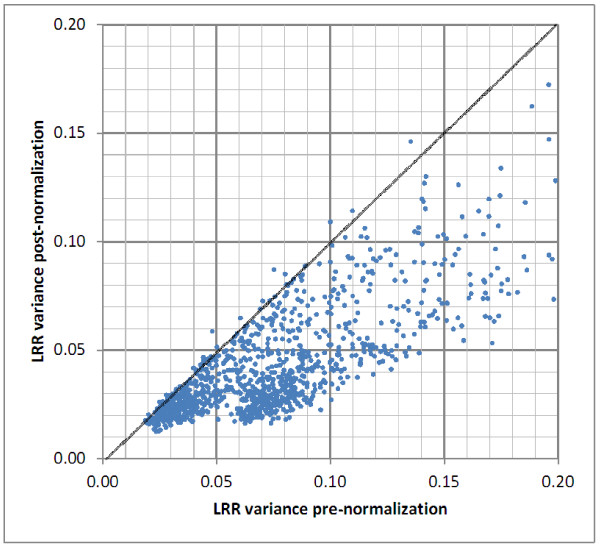
**The residual LRR variance prior to and after adjustment for C + G and CpG content.** The log probe intensity ratio (LRR) values for each SNP or CNV assay provides data on probe intensity relative to that of the estimated genotype-specific cluster location. We implemented a method similar to that described in Staaf *et al *. [29] to re-estimate LRR after a quantile-normalization, with an enhanced multiple linear regression model, incorporating within-chip signal re-scaling terms and a polynomial correction for GC and CpG waves. This scatterplot shows the pre-normalization LRR variance against the LRR variance post-normalization.

To explore the TUF phenomenon genome wide, we utilized data from 1252 Illumina genotyping runs [20] and, on a sample by sample basis, regressed the log probe intensity ratio (LRR) on eight C + G and eight CpG terms for genomic window sizes of 50 bp to 1 Mbp. The residual variance prior to and after adjustment for C + G and CpG is shown in Figure 6. The samples that showed the largest correlations with the C + G and CpG terms, measured by the proportion of LRR variance explained, involved C + G content size windows of 0.1 - 10 kb (Z scores greater than 30 or less than −30), and were also observed with a lower significance with CpG content and other window sizes. We then experimentally tested the amplification behavior of DNAs for samples for which the correlation was extreme (24 positive and 19 negative), plus 11 other DNAs where no significant correlation was apparent, using two PRT assays (2n13 and 8n6). A strong statistical association was seen between PRT performance and the per sample extreme behavior on the Illumina platform when considering the smaller size windows (0.1 kb for C + G; p = 0.0001 and for the 0.5 to 5 kb range for CpG; p between 0.01 and 0.00085), as shown in Table 2. This fits perfectly with the notion that many particularly C + G rich elements (including CpG islands) across the genome influence the efficiency of analysis of surrounding contiguous sequences by severely hindering DNA denaturation.

**Table 2:** Association between PRT performance and Illumina Infinium intensity correlations with C + G and CpG for 54 samples

	**C + G**		**CpG**	
**Window**	**Correlation**	**Spearman rank**	**Correlation**	**Spearman rank**
**Size**	**p-value**	**p-value**	**p-value**	**p-value**
1 mb	0.7	0.8	0.37	0.59
100 kb	**0.04**	**0.05**	**0.002**	**0.007**
50 kb	**0.04**	0.13	0.41	0.4
10 kb	0.36	0.29	0.49	0.28
5 kb	0.39	0.36	**0.00085**	**0.013**
1 kb	0.66	0.52	**0.033**	**0.023**
500 bp	0.55	0.51	**0.016**	**0.008**
100 bp	**0.0001**	**0.0002**	**0.066**	**0.028**

These observations imply that it should be possible to bioinformatically predict and partially correct for the effects of TUF areas of the genome and for other phenomena that have been observed to induce similar C + G correlated effects. Diskin *et al*. [21] demonstrate that C + G-correlated intensity fluctuations (waves) are present in both Illumina and Affymetrix whole-genome SNP microarrays and that C + G content in 1 Mb windows are highly correlated with intensity (both positively and negatively) with the amplitude determined by the degree that DNA quantity/concentration deviated from the vendor’s recommended level. Efficiency of PCR amplification of short DNA fragments (<200 bp) has also been shown to be affected by local C + G-content and some suggestions have been made on how to predict and compensate for such effects [22].

### Artificial generation or repair of DNA nicking/fragmentation

**Figure 7:**
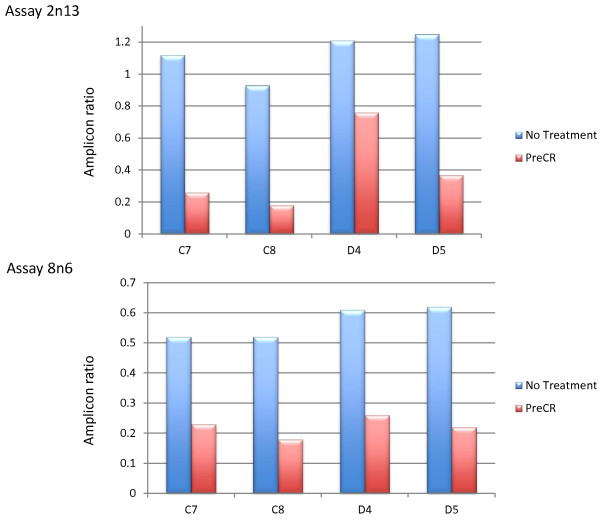
**Ligase treatment drastically reduces PCR efficiency in TUF regions.** The charts indicate the amplicon product ratios for two PRT assays for four ‘old’ DNA samples (C7, C8, D4, D5) that were untreated (blue) and treated with PreCR (red), which includes a ligase to repair ssDNA nicks. Treated samples have greatly reduced amplification efficiency at the test amplicon in the ‘TUF’ region compared to the untreated DNA samples.

**Figure 8:**
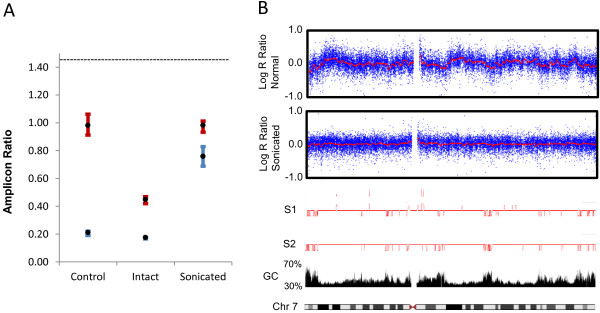
**Sonicated DNA improves the quality of WGA and Illumina Infinium genotyping.** (**A**) This graph shows test:reference product ratios (Y-axis) for PRT assay 2n13 performed using equal amounts of various input DNAs as labelled (X-axis). All PRT reactions were run in quadruplicate, with maximum and minimum plotted as error bars. The dotted horizontal line at 1.43 indicates the ratio that would be produced if the test and reference amplicons amplified with exactly equal efficiency. ‘Control’ indicates that the PRT employed freshly prepared genomic DNA. ‘Intact’ indicates that the same genomic sample was first subjected to WGA using the MDA method (QIAGEN Repli-g Mini kit applied to 50 ng of DNA). ‘Sonicated’ indicates that the same genomic sample was first sonicated to less than 1 kb average size and WGA processed. The blue data points show data produced by the above regimes, whereas the red data points are from an equivalent experiment where the DNA was additionally digested with *Nco*I immediately prior to inclusion in the PRT reactions. As indicated in Figure 4, *Nco*I cuts the genomic DNA just upstream of the test sequence target region, and separates it from a nearby region of high C + G content. The data points for the ‘intact’ column demonstrate that after WGA the test locus is still subject to reduced amplification efficiency. Importantly, correction by digestion is substantially reduced compared to the control. Sonication prior to WGA dramatically enhances amplification to almost the efficiency of the references locus even without correction by digestion. (**B**) Using chromosome 7 as a typical example, log R ratio plots (a measure of relative signal strength) are shown for Illumina Infinium genotyping data generated by assaying a freshly prepared intact genomic DNA sample (log R ratio plot in the upper box) and from a portion of that sample sonicated to 0.3 – 3 kbp in size (log R ratio plot in the second box). The data tracks below these boxes show the apparently reduced signal strength regions (as copy number inferences) generated on the same platform for two poorly performing DNA samples (those mentioned in Figure 1), the C + G content and CpG island maps, and the chromosome 7 ideogram.

The discovery and descriptive elucidation of TUF allows us to draw several important practical conclusions. Critically, the experimental impact of the phenomenon on any particular DNA sample will depend upon how nicked or fragmented that sample is, because the density of strand discontinuities will affect the probability of any particular DNA sequence being separated from C + G rich elements. Counter-intuitively, this implies that newly prepared, highly intact DNAs will be most vulnerable to TUF induced problems, whereas older and/or more degraded samples will be less affected. In support of this, we artificially ‘rejuvenated’ nicked, old DNAs by ligase treatment (PreCR by NEB), and found that this made them far more susceptible to TUF as measured by our PRT assays (Figure 7). Conversely, by artificially introducing nicks and breaks into DNA one can overcome the effect of TUF (as seen above for restriction enzyme digestion, Figure 5), ensuring highly uniform assay behaviour across genome regions and samples. This benefit of DNA fragmentation was also demonstrated for WGA (Multiple displacement amplification [23,24] - which is often applied before genotyping or sequencing), and for the overall process of Illumina Infinium genotyping (Figure 8). In both cases, sonication of the sample prior to each protocol greatly improved the quality and uniformity of the results.

## Conclusions

In summary, our description of TUF represents the important recognition of a phenomenon relevant to many regions of the genome, thus impacting in a sample dependant manner the conduct of genome-wide studies of distinct types of genetic variation in relation to human diseases/traits. For example, it may well be practically relevant in Copy Number Variation (CNV) research and the use of next generation sequencing, where assay behavior can be unpredictable [25-28]. Further work will be required to fully understand the biochemical basis of the TUF regions in order to optimally develop protocols and approaches for large scale genomic analyses. Knowledge of the TUF phenomenon and ways to overcome its deleterious consequences should provide investigators with a more nuanced approach towards handling issues related to C + G content and its effect upon assay robustness and efficiency.

## Methods

### Human genomic DNA samples

DNA donors for Southern Blotting and PRT analysis of TUF regions were of north European origin, and had given informed consent with ethical approval from the Leicestershire, Northamptonshire and Rutland Research Ethics Committee (LNRREC Ref. No. 6659 UHL). DNA was prepared from fresh blood as follows. 20 ml whole blood was centrifuged at 1300 g at 4°C for 15 minutes. The buffy coat was extracted and incubated at 37°C in 15 ml lysis buffer (10 mM Tris-Cl (pH 8.0) 0.1 M EDTA (pH 8.0) 0.5% w/v SDS) for 1 hour. Proteinase K (final concentration 100 μg/ml) was added and mixed gently followed by incubation at 50°C overnight. After allowing to cool to room temperature an equal volume of phenol equilibrated with 0.1 M Tris HCl and mixed slowly on a Stuart Rotator SB3 for 10 mins. The phases were separated by centrifugation at 5600 g for 15 min. The aqueous phase was transferred to a fresh tube and the phenol extraction repeated twice. To the final aqueous phase 1/10^th^ volume 5 M Ammonium Acetate and 2 volumes of 100% Ethanol were added. Samples were mixed very slowly and carefully by inversion. The precipitated DNA was spooled using a glass hook and dried briefly and dissolved in water to a final concentration of 200 ng/μl. DNA quality and quantity was assessed by gel electrophoresis and on the NanoDrop ND-8000 spectrophotometer.

### Paralogue ratio test (PRT)

PRTs were designed according to information from Armour *et al.,*[14]. All PRT oligonucleotide primers are described in Table 1. 10 μl PRT PCRs contained 1 x PCR buffer (75 mM Tris HCl (pH8.8), 20 mM (NH4)_2_SO_4_, 0.01% v/v Tween) (Abgene, Epsom, Surrey, UK), 1.5 mM MgCl_2_ (Abgene), 0.15 μM of each primer (Biomers), 0.2 mM dNTPs (Promega), 0.3 U *Taq* polymerase (Kapa Biosystems, Boston, MA, USA) and 10 to 25 ng DNA. PCR were initially heated to 94°C for 30 seconds, and then heated for 25 to 35 cycles as follows: 94°C for 30 seconds; annealing temperature for 30 seconds; 72°C for 1 minute. A final extension was carried out at 72°C for 5 minutes. Where required, restriction enzyme digests were performed to allow visualisation of similar sized PRT products. On using additives (DMSO up to 50%, betaine up to 2 M) the optimal annealing temperature was re-optimised for each assay. Recommended PCR conditions for TUF regions are 1.5 M betaine, 5U/μl Taq polymerase, 0.01U/μl pfu enzyme and use of 98°C denaturing temperature in all cycles. Higher concentrations of betaine may be appropriate for individual PCRs.

### Agarose gel peak height quantification

Gels were documented using a GBOX HR, Gel documentation system (Syngene, Cambridge, Cambridgeshire, UK) using the EDR function and the maximum resolution settings (5.52 M pixels). Peaks were identified and peak heights quantified using the Gene Tools programme version 4.00 (A) (Syngene). For peak height analysis, the rolling disc method (diameter = 30 pixels) was used to determine peak base line.

### Pre-PCR heat denaturation

High temperature denaturing was performed in a 96 well format heat block set to the desired temperature. Sierra Antifreeze/coolant (Peak performance products, Northbrook, IL, USA) was used to maintain a liquid contact between the tubes, thermometer and heat block. The DNA was denatured in either water or in buffered conditions (1 x PCR buffer, as above) in tubes with the lids sealed tightly with Nescofilm to prevent evaporation at temperatures greater than 100°C. Samples were heated for 1 minute and snap cooled on ice for 5 minutes. Samples were stored at −20°C and thawed on ice prior to use.

### Sonication of DNA

Aliquots of genomic DNA (200 ng/μl) were sonicated for 30 second intervals (with a 30 second gap), using a Bioruptor (Diagenode, Liège, Belgium) until the desired size range (0.3 to 3.0 kbp) was reached (visualised by agarose gel electrophoresis).

### Adapted illumina protocol

Using conditions recommended by Illumina, 200 ng samples of genomic DNA (with or without pre-processing as necessary for each experiment) were hybridised to human370CNV Infinium HD BeadChips (Illumina INC, San Diego, CA, USA).

### Whole genome amplification

Whole genome amplification was performed using the REPLI-g Mini Kit (Qiagen) to amplify a range of masses of human genomic DNA to generate >8 μg of DNA. Samples were prepared using the isothermal amplification reaction in PCR tubes incubated at 30°C for 16 hours and 65°C for 3 minutes in a thermal cycler. Amplified products were quantified using a NanoDrop spectrophotometer and visualised on a 0.8% LE agarose gel with Ethidium Bromide.

### Restriction enzyme digestion for southern blotting

Six μg of genomic DNA was digested using selected enzymes supplied by New England Biolabs (NEB) (Hitchin, Hertfordshire, UK) under the conditions recommended by the supplier with the addition of 4 mM Spermidine pH 7.4. Double digests were performed in the most suitable buffer, and the quantity of the least active enzyme per reaction was doubled if required.

### DNA denaturing prior to southern blotting

Heat denaturation was performed in a water-bath at 100°C for either for 40 seconds to 4 minutes as stated. Samples were snap cooled on ice for 5 minutes prior to gel electrophoresis.

Alkaline denaturation was performed by addition of 0.4 M NaOH to 0.32 M (~ 240 μl added to 54 μl of sample), and incubation at room temperature for 10 minutes. 1 M Tris Hcl (pH 8) was added to 0.02 M prior to neutralisation (pH 8 to 8.5) with 0.4 M HCl. Samples were ethanol precipitated and dissolved in distilled water.

### Southern blotting and hybridisation

Digested DNA was run at 3 V/cm in 0.7% agarose gels (LE agarose, Seakem. 1 X TAE (4.84 g Tris base, 11.4 ml glacial acetic acid, 3.7 g EDTA pH 8.0 per litre)). The resulting gels were soaked twice in denaturing solution (1.5 M NaCl, 0.5 M NaOH) for 30 minutes, and twice in neutralising solution (0.5 M Tris pH 7.2, 1 M NaCl) for 30 min. The denatured DNA was transferred onto uncharged nylon membranes (MAGNA, Nylon, Transfer Membrane, 0.45 Micron; GE Water & Process Technologies, Trevose, PA, USA) using 10X SSC as the transfer buffer and fixed to the membranes by baking at 80°C in a Sanyo MOV drying oven (Sanyo E&E Europe BV, Biomedical Division, Loughborough, Leicestershire, UK), for 1 hour.

PCR amplified probes (Table 1) were purified using a Qiagen MinElute PCR purification kit (Qiagen). 75 ng of probe was labelled for 15 minutes with α-32P –dCTP (Perkin Elmer, Waltham, MA USA) using the Rediprime II random prime labelling system (Amersham Biosciences, Little Chalfont, Buckinghamshire, UK), purified using ILLUSTA NICK Columns Sephadex DNA grade (GE Healthcare, Little Chalford, Buckinghamshire, UK), and eluted in 400 μl column wash (1 x TE, 0.1% w/v SDS). 75 μg of human Cot I DNA (Invitrogen, Paisley, Renfrewshire, UK) was added prior to denaturation at 100°C for 6 minutes and snap cooling on ice for 5 minutes.

Hybridisation was performed in 20 ml Church buffer (0.5 M sodium phosphate, pH 7.2, 7% SDS, 1 mM EDTA, 1% BSA ) with 2 mg heat denatured (100°C for 5 min, ice for 5 min) salmon sperm DNA. Pre-hybridisation was performed at 65°C in a rolling bottle for 2 hours prior to hybridisation for 10 hours. Hybridised blots were washed for 10 min at 65°C in 0.1 x SSC, 0.1% SDS. Counts were recorded using a phosphoimager screen (Amersham Biosciences) for between 12 and 60 hours. Further washing at 68°C or 72°C depending on the number of background counts.

### Regression analysis of LRR and G + C/CpG content for varying window sizes

The log probe intensity ratio (LRR) value for each SNP or CNV assay provides data on probe intensity relative to that of the estimated genotype-specific cluster location. LRR values estimated by the Genome Studio software were corrected for bias due to the properties of the assay chemistry and fluorescent dyes used in the probes. We implemented a method similar to that described by Staaf *et al*. [29] to re-estimate LRR after applying quantile-normalization, with an enhanced multiple linear regression model, incorporating within-chip signal re-scaling terms and a polynomial correction for GC and CpG waves. The correction model is an extension to the method described in Diskin *et al.*[21] with terms for multiple window sizes for proportion of GC and CpG content around the genomic location of each set of probes. GC and CpG terms in the regression model are the proportion of GC and CpG content for window sizes (in bp) of 50, 100, 500, 1 k, 10 k, 50 k, 100 k, 250 k, and 1 M centered around the genomic location of each assay, based on locations annotated in the Illumina manifest files and sequence context based on the NCBI build 36 reference genome sequence. This model is estimated per sample, as the phenomenon is modulated by TUF, the concentration of the DNA input, and possibly other factors. The final LRR was re-computed using the resulting quantile-normalized and GC/CpG corrected values as shown in Peiffer *et al.*[30]. The reduction in variance of the LRR values is shown in Figure 6.

## Abbreviations

TUF: Thermodynamically ultra-fastened; DNA: Deoxyribonucleic acid; WGA: Whole genome amplification; SNP: Single nucleotide polymorphism; PCR: Polymerase chain reaction; PRT: Paralogue ratio test; DMSO: Dimethyl sulphoxide; CNV: Copy number variation.

## Competing interests

The authors declare that there are no competing interests.

## Authors’ contributions

CDV participated in study design and coordination, critical analysis of results, performed bioinformatic and statistical comparisons between datasets and drafted the manuscript. PJF planned and performed Southern Blots, PRTs, WGA and Illumina Infinium genotyping experiments and aided in analysis of results. KJ performed statistical analysis of Illumina Infinium raw intensity data and drafted the manuscript. OL carried out alignment of Illumina data with genome features. SJ and ML collected DNA samples and performed data analysis of Illumina genotyping. DA analysed genotyping datasets. RRV performed Southern Blots and interpreted data. IG performed Illumina Infinium genotyping and participated in analysis of results. SJC participated in participated in experiment design, critical analysis of results and drafting of manuscript. AJB conceived the study, participated in its design and coordination, critical analysis of results and drafting of manuscript. All authors read and approved the final manuscript.

## References

[B1] Pugh TJ Delaney AD Farnoud N Flibotte S Griffith M Li HI Qian H Farinha P Gascoyne RD Marra MA Impact of whole genome amplification on analysis of copy number variants Nucleic Acids Res 2008 36 e80 10.1093/nar/gkn378 18559357 PMC2490749

[B2] Dickson PA Montgomery GW Henders A Campbell MJ Martin NG James MR Evaluation of multiple displacement amplification in a 5 cM STR genome-wide scan Nucleic Acids Res 2005 33 e119 10.1093/nar/gni126 16055919 PMC1182175

[B3] Bergen AW Qi Y Haque KA Welch RA Chanock SJ Effects of DNA mass on multiple displacement whole genome amplification and genotyping performance BMC Biotechnol 2005 5 24 10.1186/1472-6750-5-24 16168060 PMC1249558

[B4] Cunningham JM Sellers TA Schildkraut JM Fredericksen ZS Vierkant RA Kelemen LE Gadre M Phelan CM Huang Y Meyer JG Pankratz VS Goode EL Performance of amplified DNA in an Illumina GoldenGate BeadArray assay Cancer epidemiology biomarkers prevention a publication of the American Association for Cancer Research cosponsored by the American Society of Preventive Oncology 2008 17 1781 1789 10.1158/1055-9965.EPI-07-2849 PMC2732190 18628432

[B5] Berthier-Schaad Y Kao WHL Coresh J Zhang L Ingersoll RG Stephens R Smith MW Reliability of high-throughput genotyping of whole genome amplified DNA in SNP genotyping studies Electrophoresis 2007 28 2812 2817 10.1002/elps.200600674 17702060

[B6] Usdin K Woodford KJ CGG repeats associated with DNA instability and chromosome fragility form structures that block DNA synthesis in vitro Nucleic Acids Res 1995 23 4202 4209 10.1093/nar/23.20.4202 7479085 PMC307363

[B7] McDowell DG Burns NA Parkes HC Localised sequence regions possessing high melting temperatures prevent the amplification of a DNA mimic in competitive PCR Nucleic Acids Res 1998 26 3340 3347 10.1093/nar/26.14.3340 9649616 PMC147702

[B8] Benita Y Oosting RS Lok MC Wise MJ Humphery-Smith I Regionalized GC content of template DNA as a predictor of PCR success Nucleic Acids Res 2003 31 e99 10.1093/nar/gng101 12907751 PMC169991

[B9] Baskaran N Kandpal RP Bhargava AK Glynn MW Bale A Weissman SM Uniform amplification of a mixture of deoxyribonucleic acids with varying GC content Genome Res 1996 6 633 638 10.1101/gr.6.7.633 8796351

[B10] Howell R Usdin K The ability to form intrastrand tetraplexes is an evolutionarily conserved feature of the 3’ end of L1 retrotransposons Mol Biol Evol 1997 14 144 155 10.1093/oxfordjournals.molbev.a025747 9029792

[B11] Simpson JR Nagle WA Bick MD Belli JA Molecular Nature of Mammalian Cell DNA in Alkaline Sucrose Gradients Proc Natl Acad Sci USA 1973 70 3660 3664 10.1073/pnas.70.12.3660 4519655 PMC427301

[B12] Russell AP Holleman DS The thermal denaturation of DNA: average length and composition of denatured areas Nucleic Acids Res 1974 1 959 978 10.1093/nar/1.8.959 10793728 PMC343404

[B13] Hollox EJ Huffmeier U Zeeuwen PLJM Palla R Lascorz J Rodijk-Olthuis D Van De Kerkhof PCM Traupe H De Jongh G Den Heijer M Reis A Armour JAL Schalkwijk J Psoriasis is associated with increased beta-defensin genomic copy number Nat Genet 2008 40 23 25 10.1038/ng.2007.48 18059266 PMC2447885

[B14] Armour JAL Palla R Zeeuwen PLJM Den Heijer M Schalkwijk J Hollox EJ Accurate, high-throughput typing of copy number variation using paralogue ratios from dispersed repeats Nucleic Acids Res 2007 35 e19 10.1093/nar/gkl1089 17175532 PMC1807953

[B15] Chakrabarti R Schutt CE The enhancement of PCR amplification by low molecular-weight sulfones Gene 2001 274 293 298 10.1016/S0378-1119(01)00621-7 11675022

[B16] Frackman BS Kobs G Simpson D Storts D Corporation P Betaine and DMSO: Enhancing Agents for PCR Promega Notes 1998 65 9 12

[B17] Oshima RG Single-stranded DNA binding protein facilitates amplification of genomic sequences by PCR Biotechniques 1992 13 188 1382464

[B18] Rees WA Yager TD Korte J Von Hippel PH Betaine can eliminate the base pair composition dependence of DNA melting Biochemistry 1993 32 137 144 10.1021/bi00052a019 8418834

[B19] Geiduschek EP On the factors controlling the reversibility of DNA denaturation J Mol Biol 1962 4 467 487 10.1016/S0022-2836(62)80103-X 13897515

[B20] Landi MT Chatterjee N Yu K Goldin LR Goldstein AM Rotunno M Mirabello L Jacobs K Wheeler W Yeager M Bergen AW Li Q Consonni D Pesatori AC Wacholder S Thun M Diver R Oken M Virtamo J Albanes D Wang Z Burdette L Doheny KF Pugh EW Laurie C Brennan P Hung R Gaborieau V McKay JD Lathrop M A Genome-wide Association Study of Lung Cancer Identifies a Region of Chromosome 5p15 Associated with Risk for Adenocarcinoma Am J Hum Genet 2009 85 679 691 10.1016/j.ajhg.2009.09.012 19836008 PMC2775843

[B21] Diskin SJ Li M Hou C Yang S Glessner J Hakonarson H Bucan M Maris JM Wang K Adjustment of genomic waves in signal intensities from whole-genome SNP genotyping platforms Nucleic Acids Res 2008 36 e126 10.1093/nar/gkn556 18784189 PMC2577347

[B22] Aird D Ross MG Chen W-S Danielsson M Fennell T Russ C Jaffe DB Nusbaum C Gnirke A Analyzing and minimizing PCR amplification bias in Illumina sequencing libraries Genome Biol 2011 12 R18 10.1186/gb-2011-12-2-r18 21338519 PMC3188800

[B23] Blanco L Bernad A Lázaro JM Martín G Garmendia C Salas M Highly efficient DNA synthesis by the phage phi 29 DNA polymerase. Symmetrical mode of DNA replication J Biol Chem 1989 264 8935 8940 2498321

[B24] Lizardi PM Huang X Zhu Z Bray-Ward P Thomas DC Ward DC Mutation detection and single-molecule counting using isothermal rolling-circle amplification Nat Genet 1998 19 225 232 10.1038/898 9662393

[B25] Hert DG Fredlake CP Barron AE Advantages and limitations of next-generation sequencing technologies: a comparison of electrophoresis and non-electrophoresis methods Electrophoresis 2008 29 4618 4626 10.1002/elps.200800456 19053153

[B26] Brockman W Alvarez P Young S Garber M Giannoukos G Lee WL Russ C Lander ES Nusbaum C Jaffe DB Quality scores and SNP detection in sequencing-by-synthesis systems Genome Res 2008 18 763 770 10.1101/gr.070227.107 18212088 PMC2336812

[B27] Bentley DR Balasubramanian S Swerdlow HP Smith GP Milton J Brown CG Hall KP Evers DJ Barnes CL Bignell HR Boutell JM Bryant J Carter RJ Keira Cheetham R Cox AJ Ellis DJ Flatbush MR Gormley NA Humphray SJ Irving LJ Karbelashvili MS Kirk SM Li H Liu X Maisinger KS Murray LJ Obradovic B Ost T Parkinson ML Pratt MR Accurate whole human genome sequencing using reversible terminator chemistry Nature 2008 456 53 59 10.1038/nature07517 18987734 PMC2581791

[B28] Marioni JC Thorne NP Valsesia A Fitzgerald T Redon R Fiegler H Andrews TD Stranger BE Lynch AG Dermitzakis ET Carter NP Tavaré S Hurles ME Breaking the waves: improved detection of copy number variation from microarray-based comparative genomic hybridization Genome Biol 2007 8 R228 10.1186/gb-2007-8-10-r228 17961237 PMC2246302

[B29] Staaf J Vallon-Christersson J Lindgren D Juliusson G Rosenquist R Höglund M Borg Å Ringnér M Normalization of Illumina Infinium whole-genome SNP data improves copy number estimates and allelic intensity ratios BMC Bioinforma 2008 9 409 10.1186/1471-2105-9-409 PMC2572624 18831757

[B30] Peiffer DA Le JM Steemers FJ Chang W Jenniges T Garcia F Haden K Li J Shaw CA Belmont J Cheung SW Shen RM Barker DL Gunderson KL High-resolution genomic profiling of chromosomal aberrations using Infinium whole-genome genotyping Genome Res 2006 16 1136 1148 10.1101/gr.5402306 16899659 PMC1557768

